# “The Clock Is Ticking”: The Timely Management of a Painful Skin Rash in a Seventy-Year-Old Woman

**DOI:** 10.1155/2014/641058

**Published:** 2014-02-23

**Authors:** Susan Thomas, Folashade Omole, Vijaykumar G. Patel, Michelle L. Nichols

**Affiliations:** ^1^Department of Family Medicine, Morehouse School of Medicine, 1513 East Cleveland Avenue, Building 100, Suite 300A, Atlanta, GA 30344, USA; ^2^Department of Surgery, Morehouse School of Medicine, 1513 East Cleveland Avenue, Building 100, Suite 300A, Atlanta, GA 30344, USA

## Abstract

Necrotizing fasciitis is an uncommon but a potentially fatal condition and can affect any part of the body. Most patients have pre-existing conditions that render them susceptible to infection, although etiology is unclear. Diagnosis is primarily clinical and is often delayed because of the unfamiliarity of the condition among clinicians. Management consists of immediate resuscitation, early surgical debridement, and administration of broad spectrum intravenous antibiotics. We report a case of a 70 year old woman who presented with a painful erythematous rash, was admitted as a case of cellulitis, later developed worsening of symptoms and septic shock, and was diagnosed as necrotizing fasciitis.

## 1. Introduction

Necrotizing fasciitis is an uncommon disease that results in morbidity and mortality if not treated early. Most patients who develop necrotizing fasciitis have preexisting conditions that render them susceptible to infection. Advanced age, diabetes mellitus, chronic renal failure, peripheral vascular disease, and drug misuse seem to be risk factors [1]. We report a case of necrotizing fasciitis in a diabetic patient who presented with redness and pain of right thigh.

## 2. Case Report

**Figure 1:**
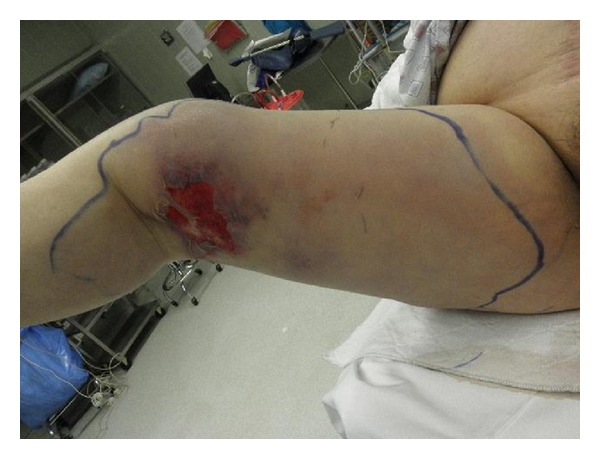
Preoperative photograph on the day of admission showing rapidly progressive painful extensive erythema, blistering, ulceration, edema, and skin necrosis of the right leg due to necrotizing soft tissue infection.

**Figure 2:**
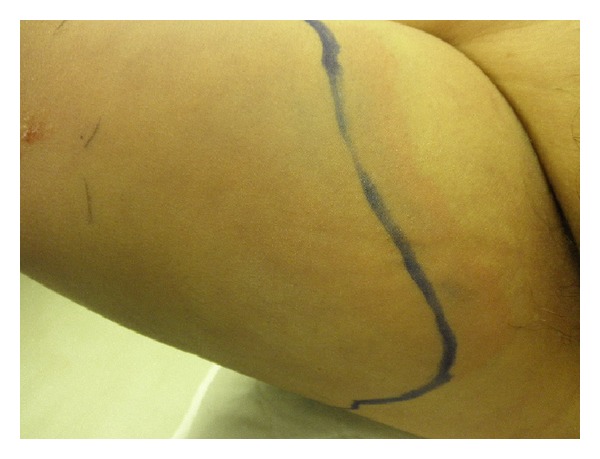
Photograph showing line of demarcation of erythema after admission and subsequent rapid progression of erythema within 1 hour. The rapid spread of necrotizing infection is facilitated by the enzymes and toxins produced by the organisms and is one of the hallmarks for clinical diagnosis.

**Figure 3:**
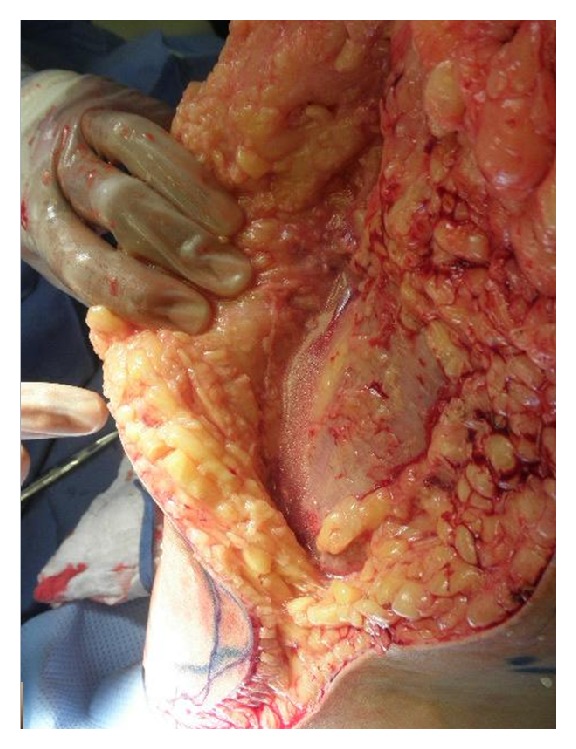
Intraoperative photograph showing deep necrotizing infection involving subcutaneous tissue along superficial and deep facial planes with watery purulent fluid adjacent to deep fascia and underlying muscles.

**Figure 4:**
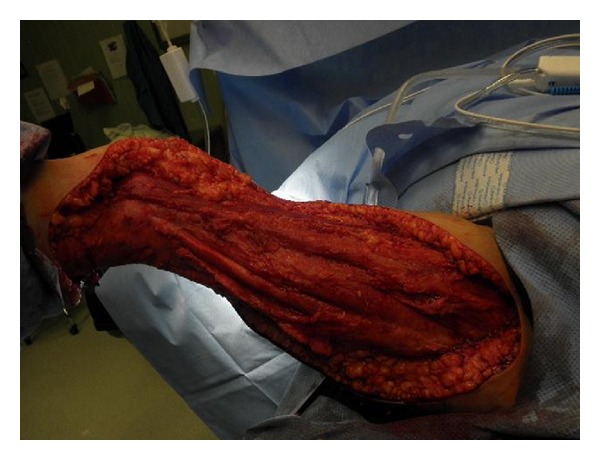
Intraoperative photograph showing aggressive surgical debridement of all necrotic and infected tissues involving the right leg.

A 70-year-old woman with a history of noninsulin requiring diabetes mellitus, hypertension, hyperlipidemia, and chronic atrial fibrillation on warfarin therapy, returning home from a cruise to Bahamas, presented to the emergency room. She reported a one-day history of a 10/10 pain in right thigh and leg, subjective fever, and nausea. Physical examination revealed blood pressure 133/89 mm Hg, heart rate 94/minute, respiratory rate 18/minute, and temperature 101.30 F. Oxygen saturation was 96% on room air. There was marked erythema with induration of the skin involving the posterior thigh and leg along with the popliteal fossa measuring about 12 cm in length. Laboratory and radiological investigation revealed a white blood cell count of 19.7/*μ*L with 85% neutrophils, D-dimer of 2.9, and an international ratio (INR) of 3.16. Doppler ultrasound of the right lower extremity was negative for deep venous thrombosis. She was admitted with the diagnosis of cellulitis and started on intravenous clindamycin and fluids. On hospital day one, there was worsening of the lower extremity pain and erythema (Figures 1, 2, 3, and 4), with development of disseminated intravascular coagulation (DIC) and septic shock. Subsequent lab results showed sodium 144 mmol/L (millimoles/litre), potassium 4.2 mmol/L, chloride 97 mmol/L, and bicarbonate 12 mmol/L. Blood glucose was 181 mg/dL, and C-reactive protein (CRP) was elevated at 15.8 mg/L. Urea was 30 mg/dL and creatinine 2.5 mg/dL. Patient's hemoglobin and hematocrit were 9.2 g/dL and 27.4%, respectively. And other laboratory values were a lactic acid of 19.6 mmol/L, and a prothrombin time of 61.7 seconds with an INR of 5.76. The Laboratory Risk Indicator for necrotizing fasciitis (LRINEC) score was 6 in the patient. She was transferred to intensive care unit (ICU), intubated, and started on vasopressors, along with penicillin, vancomycin, and zosyn (piperacillin and tazobactam). She was resuscitated with 4 litres of normal saline, and during the course of her ICU admission, before and after debridement, she received a total of 13 units of packed red blood cells (PRBC), 9 units of fresh frozen plasma (FFP), and 4 units of platelets. Initial surgical debridement was done within six hours (see the figures) and two more over the next forty-eight hours. Patient was also hemodialyzed due to acute renal failure. Blood and wound cultures grew group A *Streptococcus* (*Streptococcus pyogenes*) sensitive to penicillin. The gross pathological specimen revealed wide excision of skin, subcutaneous tissue, and deep fascia down to the muscle from proximal thigh to mid-calf measuring 44 × 22 × 2.5 cm, with evidence of skin sloughage reddish yellow measuring 8 × 6.5 cm. With successful and early surgical debridement, appropriate antibiotic therapy, and other supportive treatments, patient's shock and DIC resolved. Patient received skin grafting at the surgical site. Tracheostomy was done due to difficulty to wean patient off the ventilator after two prolonged weeks of intubation; patient was transferred to a long-term care facility for prolonged ventilator care and rehabilitation.

## 3. Discussion

Necrotizing skin infections were first described by Jones in 1871, and “hospital gangrene” was the term used then [2]. Most patients who develop necrotizing fasciitis have preexisting conditions that render them susceptible to infection. Conditions such as advanced age, diabetes mellitus (as in our patient), chronic renal failure, peripheral vascular disease, and drug misuse seem to be risk factors [1]. However, the etiology of necrotizing fasciitis is not fully understood, and in many cases no identifiable cause can be found [3]. It occurs slightly more often in male patients [1].

### 3.1. Clinical Features

Necrotizing fasciitis can affect any part of the body, but the extremities, perineum, and the trunk are most commonly affected [4]. Most patients present with erythema, swelling, and pain in the affected site. Severe pain disproportionate to local findings, in association with systemic toxicity, should raise the suspicion of necrotizing fasciitis.

### 3.2. Diagnosis

The findings of crepitus and soft tissue air on plain radiograph are seen in 37% and 57% of patients, respectively [1]. Even though it is considered pathognomonic for the disease, the absence of crepitus should not exclude the diagnosis and therefore having a great index of suspicion is paramount [1, 4]. CT and MRI have limited roles and may delay treatment [5]. Other findings that are common in necrotizing fasciitis include leukocytosis, elevated glucose, urea, and creatinine levels. Hypoalbuminemia, acidosis, and an altered coagulation profile may also be present [1, 3, 4, 6]. Most studies have shown that necrotizing fasciitis is polymicrobial, in nature, with *Streptococcus* being the most common causative organism [1, 3, 6], although a review study revealed that group A streptococci (GAS) was isolated in only 15% of individuals with necrotizing fasciitis [7]. There has been an emergence of toxic shock strains of *Streptococcus* (group A *β* hemolytic streptococci) leading to fasciitis with organ dysfunction [1, 8]. This virulent organism received much press coverage as the “flesh eating bacterium.” Table 1 shows the Laboratory Risk Indicator for Necrotizing Fasciitis (LRINEC) score, this is used to risk stratify patients (Table 2), that is, heighten the suspicion of NF to differentiate it from other skin and soft tissue infections [2]. A score of 6 and above is considered to be of intermediate or high risk and has a positive predictive value of 92% and a negative predictive value of 96%, and therefore clinical suspicion should still trump the LRINEC score [2, 9, 10]. The “finger test” which is characterized by lack of resistance to finger dissection in normally adherent tissues done under local or general anesthesia to assess the need for surgical debridement is a quick and easy test [2]. Macroscopic findings during surgical exploration include gray necrotic tissue, lack of bleeding, thrombosed vessels, “dishwater” pus, noncontracting muscle, and a positive “finger test” result.

### 3.3. Management

The core of the treatment is early surgical debridement, which significantly improves mortality compared with delayed surgery [6]. Delaying surgical debridement more than 24 hours of admission was found to be associated with a mortality of 25% versus 6% when surgical debridement is done early [11, 12]. Further surgical exploration 24–48 hours later is mandatory to ensure that the infectious process has not extended [8]. Resuscitative and supportive care is also paramount. Principles of Treatment [2, 8, 13] are as follows:

 adequate fluid resuscitation,
 correction of electrolyte and acid-base abnormalities,
 rapid initiation of antimicrobial therapy,
 immediate surgical debridement of necrotic tissues,
 support for failing organs.

## 4. Conclusion

Necrotizing fasciitis is an uncommon, but life threatening, condition with a high associated mortality and morbidity. Prompt diagnosis is the key to a favorable outcome. The LRINEC score is useful, but the diagnosis is still primarily a clinical one, and suspicion alone warrants early surgical referral.

## Figures and Tables

**Table 1:** LRINEC scoring system to help discriminate between necrotizing and nonnecrotizing soft tissue infections [2].

Value	LRINEC score, points
C-reactive protein, mg/L	
<150	0
>150	4
WBC count, cells/mm^3^	
<15	0
15–25	1
>25	2
Hemoglobin level, g/dL	
>13.5	0
11–13.5	1
<11	2
Sodium level, mmol/L	
≥135	0
<135	2
Creatinine level, mg/dL	
≤1.6	0
>1.6	2
Glucose level, mg/dL	
≤180	0
>180	1

Goldstein et al. [2].

**Table 2:** Risk stratification of the LRINEC score according to the likelihood of necrotizing soft tissue infection (NSTI) [2].

Risk category	LRINEC score, points	Probability of NSTI, %
Low	≤5	<50
Intermediate	6-7	50–75
High	≥8	>75

Goldstein et al. [2].

## References

[1] Elliott DC, Kufera JA, Myers RAM (1996). Necrotizing soft tissue infections: risk factors for mortality and strategies for management. *Annals of Surgery*.

[2] Goldstein EJ, Anaya DA, Dellinger EP (2007). Necrotizing soft-tissue infection: diagnosis and management. *Clinical Infectious Diseases*.

[3] Singh G, Sinha SK, Adhikary S, Babu KS, Ray P, Khanna SK (2002). Necrotising infections of soft tissues-a clinical profile. *European Journal of Surgery*.

[4] Golger A, Ching S, Goldsmith CH, Pennie RA, Bain JR (2007). Mortality in patients with necrotizing fasciitis. *Plastic and Reconstructive Surgery*.

[5] Wysoki MG, Santora TA, Shah RM, Friedman AC (1997). Necrotizing fasciitis: CT characteristics. *Radiology*.

[6] McHenry CR, Piotrowski JJ, Petrinic D (1995). Determinants of mortality for necrotizing soft-tissue infections. *Annals of Surgery*.

[7] Eke N (2000). Fournier’s gangrene: a review of 1726 cases. *British Journal of Surgery*.

[8] Hasham S, Matteucci P, Stanley PRW, Hart NB (2005). Necrotising fasciitis. *British Medical Journal*.

[9] Wong C-H, Khin L-W, Heng K-S, Tan K-C, Low C-O (2004). The LRINEC (laboratory risk indicator for necrotizing fasciitis) score: a tool for distinguishing necrotizing fasciitis from other soft tissue infections. *Critical Care Medicine*.

[10] Wilson MP, Schneir AB (2013). A case of necrotizing fasciitis with a LRINEC score of zero: clinical suspicion should trump scoring systems. *The Journal of Emergency Medicine*.

[11] Lille ST, Sato TT, Engrav LH, Foy H, Jurkovich GJ (1996). Necrotizing soft tissue infections: Obstacles in diagnosis. *Journal of the American College of Surgeons*.

[12] Wong C-H, Chang H-C, Pasupathy S, Khin L-W, Tan J-L, Low C-O (2003). Necrotizing fasciitis: clinical presentation, microbiology, and determinants of mortality. *Journal of Bone and Joint Surgery A*.

[13] Guo-Wei T, Hwabejire JO, Min-Jie J (2013). Multidisciplinary intensive care in extensive necrotizing fasciitis. *Infection*.

